# Iododerma Following Radioactive Iodine Therapy in Thyroid Cancer: Insights From 2 Cases

**DOI:** 10.1210/jcemcr/luaf056

**Published:** 2025-04-10

**Authors:** Hawra Kamal, Julie Samantray

**Affiliations:** Division of Endocrinology, Wayne State University/Detroit Medical Center, Detroit, MI 48201, USA; Division of Endocrinology, Wayne State University/Detroit Medical Center, Detroit, MI 48201, USA

**Keywords:** iododerma, radioactive iodine ablation, thyroid cancer

## Abstract

Radioactive iodine ablation is commonly used to treat thyroid diseases. However, despite its efficacy, it has inherent risks and complications. One such complication is iododerma. This rare dermatological condition triggered by iodine exposure has been infrequently documented in association with radioactive iodine therapy for thyroid diseases. Here, we present 2 cases of iododerma following radioactive iodine ablation for thyroid cancer. In the first case, a 38-year-old female developed facial swelling and red blotchy rashes accompanied by papules on the left upper eyelid. Despite initial worsening of symptoms, the patient improved after prednisone treatment. In the second case, a 71-year-old male with metastatic follicular thyroid cancer received iodine-131 therapy after levothyroxine withdrawal for pulmonary metastasis. Approximately 1 week posttherapy, he developed a nontender, nonpruritic rash on the extremities and anterior abdomen, which spontaneously resolved without intervention. Iododerma presents diagnostic challenges because of its rarity and diverse cutaneous manifestations. Although its exact pathophysiology remains unclear, it has been hypothesized to be induced by hypersensitivity reactions to and delayed clearance of iodine from the body. Physicians should be aware of this rare complication of radioactive iodine in patients with thyroid disease.

## Introduction

Radioactive iodine ablation is commonly used to treat thyroid diseases, especially differentiated thyroid cancer. It involves the administration of oral radioactive iodine, typically iodine-131 (I-131) [1]. Although the procedure is usually well tolerated, it has inherent risks and potential complications, ranging from early side effects (eg, nausea, acute sialadenitis, and iododerma) to delayed side effects (eg, chronic salivary gland disorder and secondary malignancies) [2]. Iododerma is a rare dermatological condition that is caused by exposure to iodine-containing compounds, and it can manifest in multiple cutaneous presentations. Although some cases have described iododerma after using iodinated contrast in computed tomography (CT) scans, few cases of iododerma related to radioactive iodine ablation for thyroid diseases have been reported in the literature. The development of iododerma following I-131 ablation represents a diagnostic challenge because of its rarity.

In this article, we present 2 cases of iododerma following radioactive iodine ablation for thyroid cancer, highlighting this unusual adverse effect. We seek to improve understanding and awareness of iododerma as a potential complication of radioactive iodine therapy, emphasizing the importance of early recognition and appropriate management.

## Case Presentation

### Case 1

The first case is a 38-year-old female with stage I papillary thyroid carcinoma, classified as T1N1bM0 according to the American Joint Committee on Cancer, 8th edition, presenting as a unilateral, unifocal tumor measuring 1.7 cm on the left lobe, with a single metastatic focus identified in a left level II lymph node. The patient underwent radioactive iodine ablation after levothyroxine withdrawal. Her ablation TSH level was 90.33 μIU/mL (90.33 μIU/mL), her antithyroglobulin antibody level was <0.9 IU/mL (normal reference range: 74 0.0-4.0 IU/mL), and her thyroglobulin level was 43.0 ng/mL (normal reference range: 1.3-31.8 ng/mL). The pretherapy scan showed intense tracer uptake predominantly in the left thyroid bed. The patient received 47.3 mCi for remnant ablation.

**Figure 1.**
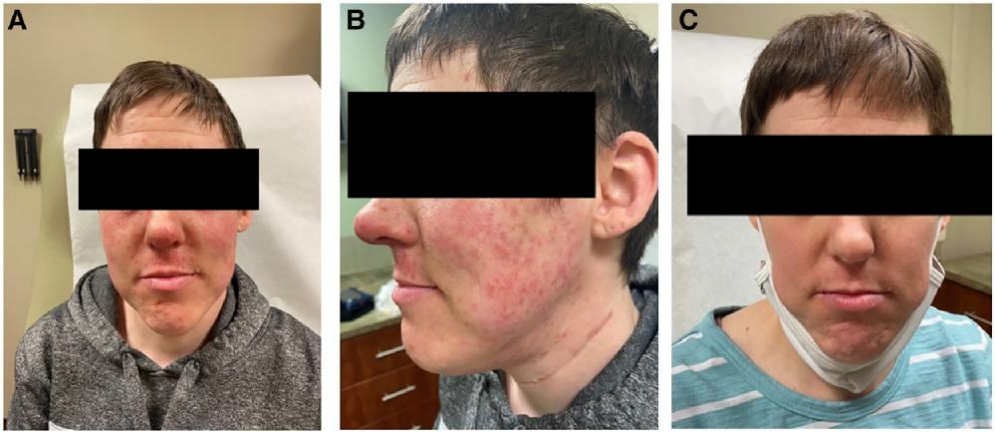
Case 1: (A) Red blotchy rashes on the face and a few scattered pink papules. (B) Image showing the patient's face before treatment. Face with erythematous patches and faint pink papules on cheeks. (C) Image showing the patient's face after treatment.

Sixteen days posttherapy, the patient developed facial swelling and periorbital edema. On the following day, she developed red, nonpruritic blotchy rashes on her face, sparing the scalp, neck, and extremities (Fig. 1). At day 18 posttherapy, additional rashes appeared on her anterior abdomen and right inner thigh on the third day.

Three weeks posttherapy, her TSH, free thyroxine, and random urine iodine levels were 19.87 μIU/mL (normal reference range: 0.45-5.33 μIU/mL), 0.85 ng/dL (10.9412 pmol/L) (normal reference range: 0.70-1.70 ng/dL; 9.01-21.88 pmol/L), and 138 μg/L (28-544 μg/L), respectively. Two months posttherapy, her TSH level was 2.69 μIU/mL.

### Case 2

A 71-year-old male was advised total thyroidectomy for an 8 cm right thyroid nodule that had been categorized as a Thyroid Imaging Reporting and Data System 4 lesion and exhibited a Bethesda III cytology, indicating a follicular lesion of undetermined significance. A positron emission tomography-CT scan performed for prostate cancer also revealed that the nodule was fluorodeoxyglucose-avid. The final pathology report showed that the thyroid nodule was a benign hyperplastic nodule.

After 2 years, the patient was found to have bilateral lung nodules on surveillance scans for his previous diagnosis of prostate cancer. A biopsy of 1 of the lung nodules was consistent with metastatic follicular thyroid cancer. Neck CT and bone scans revealed no evidence of other metastatic diseases. The patient underwent I-131 therapy (175 mCi) after levothyroxine withdrawal. At the time of ablation, laboratory tests revealed an ablation TSH of 89 μIU/mL, thyroglobulin of >450 ng/mL (normal reference range: 1.3-31.8 ng/mL), and no antithyroglobulin antibody. One week posttherapy, the patient developed a nontender, nonpruritic skin rash.

## Diagnostic Assessment

### Case 1

Clinical examination revealed erythematous patches, faint pink papules on both cheeks, axillary papules, an erythematous patch on the right upper anterior leg, and skin-colored papules on the left upper eyelid. No skin biopsy was performed.

### Case 2

**Figure 2.**
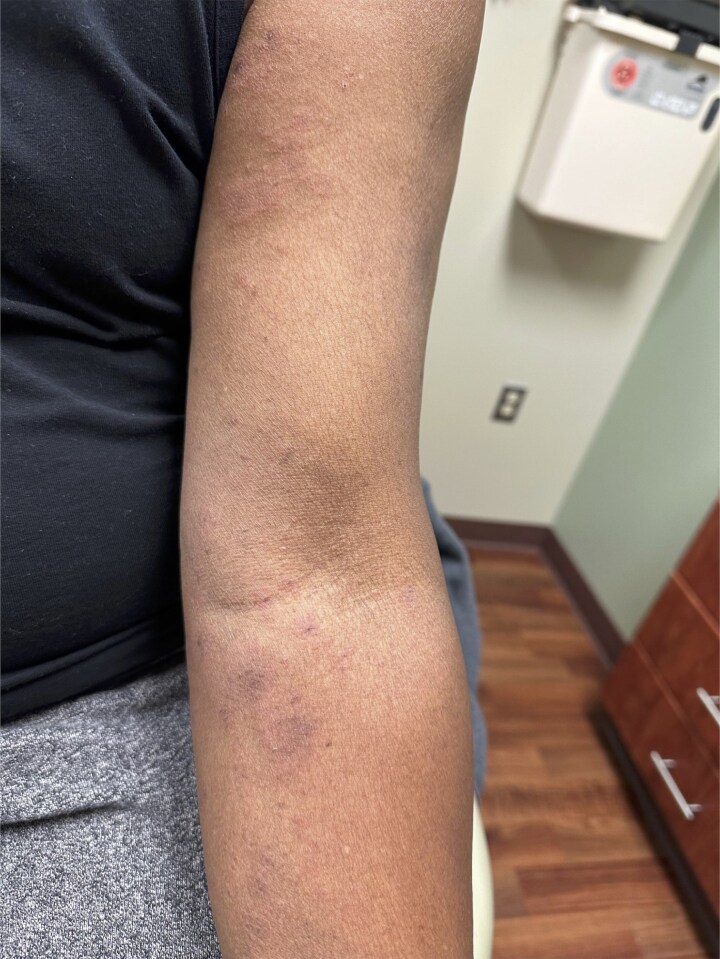
Case 2: image showing a rash affecting the left upper extremity.

Clinical examination revealed a maculopapular rash primarily on the medial aspects of the upper extremities, with minimal involvement of the anterior abdomen and thighs. The rash was nontender and nonpruritic (Fig. 2). No skin biopsy was performed.

## Treatment

### Case 1

The patient initially self-treated with diphenhydramine tablets but presented to the emergency department 4 days after onset of rashes because of worsening symptoms. Following evaluation, she was prescribed prednisone at a dose of 50 mg daily for 5 days. She was also evaluated by a dermatologist who added topical steroids until the resolution of the rashes.

### Case 2

The patient was instructed to use prednisone only if the rash worsened. However, the rash resolved on its own within 3 days without requiring medication.

## Outcome and Follow-up

### Case 1

Improvement was observed on the third day of treatment (Fig. 1). The diagnosis of iododerma was made based on the clinical presentation and timing relative to radioactive iodine therapy.

### Case 2

During follow-up visits, the patient reported that the rash had resolved without any treatment and did not recur.

## Discussion

Iododerma is a rare neutrophilic dermatosis characterized by the development of inflammatory skin lesions, which typically develop 2 to 3 weeks following iodine administration [3]. It has also been reported to be associated with various medical procedures and treatments, including the use of Lugol iodine for the preoperative preparation of thyroidectomy, iodinated contrast for CT, and topical povidone-iodine. Moreover, iododerma may be associated with systemic manifestations related to vasculitis [4]. Runge et al reported 3 cases of biopsy-proven vasculitis that they attributed to iodine toxicity [5]. These cases were observed after using iodinated contrast in CT scans and manifested as skin vesicles, bullae, and hemorrhagic papules. In addition to severe renal insufficiency, which affected the elimination time, the patients had high blood and urine iodine levels. Although the pathophysiology of iododerma remains unknown, iodide has been hypothesized to recruit to recruit polymorphonuclear leukocytes and produce circulating immune complexes leading to vasculitis [6].

Few cases of iododerma following radioactive iodine ablation of the thyroid have been reported. An inclusive review of case reports describing iododerma following radioactive iodine ablation was conducted, and the results are summarized in Table 1. The reviewed cases highlight the variability in the clinical presentation of iododerma, with onset ranging from hours to weeks following radioactive iodine ablation [7-11]. The symptoms mostly included pustular and erythematous lesions, and topical and systemic steroids were the most used treatment modality. The reported cases were predominantly females.

**Table 1.** Published cases of iododerma following RAI ablation of the thyroid

First author, year	Study type	Patient characteristics		Indication for RAI	Onset of rash	Symptoms	Treatment
		Age (years)	Sex				
Lazaga, 2011 [7]	Case report	NA	NA	Hyperfunctioning autonomous nodule	4 hours	Papulopustular rash developing on her lower extremities, progressed to the upper extremities and face	NA
Rubab, 2018 [8]	Case report	60	F	Thyroid cancer	Two weeks after the third dose	Pustular lesions and bullae on the trunk and limbs, progressed to all over the body	Topical cream (flumethasone and salicylic acid) and oral antihistamines
Vandergriff, 2011 [9]	Case report	49	F	Graves’ disease	4 days	Pustular eruption on the face, chest, and proximal extremities	Supportive care (not specified)
Sorungbe, 2019 [10]	Case report	57	F	Graves’ disease	2 weeks	Erythematous and nodular lesions	Self-limiting
Suh, 2013 [11]	Case report	40	F	Thyroid cancer	10 days	Pustular and crusted patches with erythematous and indurated bases on the face and purplish crusted desquamative plaques on the lower legs	NA

Abbreviations: NA, not available; RAI, radioactive iodine.

For example, Rubab et al described a 60-year-old female with a history of thyroid carcinoma history who developed pustular lesions on her skin after receiving multiple doses of radioactive iodine therapy. The patient had undergone thyroidectomy and received several doses of radioactive iodine over the years. Two weeks after the last dose, she developed numerous pustules and bullae on her trunk and limbs. The lesions rapidly spread but eventually resolved with treatment using a topical cream and oral antihistamines [8].

The exact pathophysiology of iododerma following radioactive iodine ablation of the thyroid remains unknown. However, various mechanisms have been hypothesized, including hypersensitivity reactions to iodine, delayed clearance of iodine from the body, and invasion of leukocytes into the skin [12]. The histopathological findings of iododerma are characterized by nonspecific changes, including a polymorphonuclear cell infiltrate with eosinophils, mast cells, plasma cells, and neutrophils in the dermis and subcutaneous tissue [13]. Moreover, the skin lesions may exhibit acute necrotizing vasculitis, and the predominant findings can include a diffuse dermal inflammatory infiltrate mostly comprising neutrophils with few plasma cells, mast cells, and eosinophils [6]. As there is no pathognomonic laboratory or histopathologic finding, the diagnosis of iododerma fundamentally depends on the clinical evaluation and exposure history, as there is no pathognomonic laboratory or histopathologic finding.

Whether iododerma is associated with the I-131 dose remains unclear. The amount of iodine in the I-131 is 20 μg per 100 mCi, which is less than that in iodinated contrast agents used in CT scans (eg, iopamidol 370 mg/L). Iododerma has been reported after hyperthyroidism treatment, which typically uses a lower dose of I-131 compared with that in thyroid cancer treatment. The rarity of this disease makes it difficult to make a conclusive inference regarding the association with the dose of I-131.

Iododerma is most commonly a self-limiting condition. However, it can be associated with systemic manifestations, including residual postinflammatory hyperpigmentation of the skin. The initial approach to managing iododerma would be the removal of the causative agent. Therefore, renal clearance after I-131 therapy plays a crucial role, and adequate hydration should be emphasized. Previous studies have highlighted the judicious use of radioiodine in thyroid cancer. Thyroxine withdrawal leads to prolonged hypothyroidism and hence slower iodine clearance. Using recombinant TSH stimulation to prepare for radioiodine therapy can mitigate this risk as the renal clearance is faster compared with that in the hypothyroid state. Moreover, the risk of recurrence of skin rashes following a subsequent I-131 therapy must be recognized. In cases where the discontinuation of I-131 therapy is not feasible, other treatment modalities may be used to manage iododerma, including pretreatment with systemic or topical corticosteroids to mitigate the development of skin lesions, or administration of topical corticosteroids may be used to relieve symptoms [4]. For severe cases, systemic steroids can be used to control inflammation and promote healing [13].

Iododerma is a rare dermatological condition associated with iodine exposure, presenting a clinical complexity that constitutes diagnostic and management challenges. The rarity of this disease is underscored by its infrequent occurrence following iodine exposure, especially in the context of thyroid disease. The clinical complexity of iododerma is apparent in its diverse cutaneous manifestations, which can encompass acneiform eruptions, pustular lesions, vegetative plaques, and bullous variants. In the absence of characteristic pathognomonic findings, a thorough clinical assessment of the patient's exposure history is essential for establishing an accurate diagnosis.

## Learning Points

- Iododerma can occur after iodine exposure through various routes including radioiodine therapy for thyroid cancer.
- The pathophysiology of iododerma remains unknown. However, several mechanisms have been hypothesized, including hypersensitivity to and delayed iodine clearance of iodine and skin infiltration by leukocytes.
- Iododerma typically improves following the cessation of iodine exposure. In some cases, corticosteroid treatment may be effective.

## Data Availability

Data sharing is not applicable to this article as no datasets were generated or analyzed during the current study.
